# COVID-19 Survivors Are Still in Need of Neuropsychiatric Support Two Years after Infection

**DOI:** 10.3390/brainsci13071034

**Published:** 2023-07-06

**Authors:** Marco Colizzi, Maddalena Peghin, Maria De Martino, Giulia Bontempo, Stefania Chiappinotto, Federico Fonda, Miriam Isola, Carlo Tascini, Matteo Balestrieri, Alvisa Palese

**Affiliations:** 1 Unit of Psychiatry, Department of Medicine (DAME), University of Udine, 33100 Udine, Italy; matteo.balestrieri@uniud.it; 2 Department of Psychosis Studies, Institute of Psychiatry, Psychology and Neuroscience, King’s College London, London SE5 8AF, UK; 3 Infectious Diseases Division, Department of Medicine (DAME), University of Udine, and Friuli Centrale University Health Service (ASUFC), 33100 Udine, Italy; maddalena.peghin@gmail.com (M.P.); bontempo.giulia@spes.uniud.it (G.B.); carlo.tascini@uniud.it (C.T.); 4 Infectious and Tropical Diseases Unit, Department of Medicine and Surgery, University of Insubria-ASST-Sette Laghi, 21110 Varese, Italy; 5 Division of Medical Statistic, Department of Medicine (DAME), University of Udine, 33100 Udine, Italy; maria.demartino@uniud.it (M.D.M.); miriam.isola@uniud.it (M.I.); 6 Department of Medicine (DAME), School of Nursing, University of Udine, 33100 Udine, Italy; stefania.chiappinotto@uniud.it (S.C.); federico.fonda@uniud.it (F.F.); alvisa.palese@uniud.it (A.P.)

**Keywords:** long COVID, post-COVID syndrome, cognition, depression, anxiety, insomnia

## Abstract

COVID-19 survivors have been reported to be at risk of long-term neuropsychiatric sequalae; however, prospective evidence in this regard is lacking. We prospectively assessed the occurrence of mental-health-domain-related symptoms over a 24-month period following COVID-19 onset in a cohort of 230 patients. Of them, 36.1% were still presenting with at least one symptom 24 months later. Across the study period, a significant reduction in overall symptoms from the onset was observed (*p* < 0.001); however, symptom prevalence was unchanged between the 12- and 24-month follow-ups across most symptomatic domains. At the 24-month follow-up, mental-health-domain-related symptoms only were higher than at the onset and were the most frequently reported symptoms. Dyspnea at the onset predicted both symptoms of psychiatric disorders (OR = 3.26, 95% CI = 1.22–8.70, and *p* = 0.019) and a lack of concentration and focus (OR = 3.17, 95% CI = 1.40–7.16, and *p* = 0.005) 24 months post-infection, with the number of comorbidities at the onset also predicting the occurrence of a lack of concentration and focus (OR = 1.52, 95% CI = 1.12–2.08, and *p* = 0.008). The findings of this study may have important public health implications, as they underlie the fact that COVID-19 survivors are still in need of neuropsychiatric support two years after infection.

## 1. Introduction

The coronavirus disease 19 (COVID-19) pandemic has had indirect detrimental effects on the general population’s mental health due to restrictions and social disruptions [1,2]. Available evidence also indicates direct effects of COVID-19, in terms of the persistence or onset of multiple symptoms, with the most common being fatigue, shortness of breath, and cognitive dysfunction. Of relevance, neuropsychiatric symptoms have also been reported, possibly reflecting phenomena occurring in much later phases among COVID-19 survivors [3]. Such conditions, referred to as ‘long COVID’ or ‘post-COVID (syndrome)’ [4], carry a significant burden for individuals’ wellbeing [5], and their longer-term effects are largely unknown. While predictors of poor mental health in the post-acute phase of the pandemic have been better elucidated [6,7], whether some patients are at an increased risk of presenting with neuropsychiatric symptoms long after infection remains to be investigated. This is of paramount importance, considering that mental health difficulties are widely expressed among the general population, and the associated enduring cognitive dysfunction, sleep problems, and somatic complaints may drastically affect people’s ability to function in everyday life [3].

## 2. Materials and Methods

### 2.1. Study Design and Participants

This study investigated the neuropsychiatric symptom trajectory among COVID-19 survivors during the 24 months following the acute COVID-19 phase, also exploring potential sociodemographic and clinical predictors of symptom occurrence. Study details, including methods and recruitment procedures, have been reported before [8]. Briefly, between 1 March and 30 May 2020, a cohort of consecutive COVID-19 adult patients, aged 18 years or older, was recruited at the university hospital of Udine, Italy, a tertiary referral hospital of about 1000 beds offering healthcare services to a population of about 350,000 people, which was identified as a regional hub for COVID-19 patients. Both inpatients and outpatients were considered eligible for inclusion in the study if they presented with a COVID-19 diagnosis.

### 2.2. Assessment

Employing a longitudinal design, information on sociodemographic, clinical, and laboratory data was collected both at baseline and at subsequent follow-up assessments up to 24 months after the disease onset. COVID-19 diagnosis was based on a positive nucleic acid amplification test (NAAT) for SARS-CoV-2 in respiratory tract specimens (confirmed diagnosis) or either laboratory or imaging findings suggestive of infection and/or positive serology (suspected diagnosis). Based on clinical presentation, as assessed with a COVID-19 disease severity scale, patients were also classified as a function of the disease severity, from asymptomatic to critical disease. Finally, patients were also classified based on their disease management, depending on whether they were deemed to receive intensive care unit (ICU), hospital ward, or outpatient-based support [8]. This report focuses on the 24-month follow-up assessment. As was performed for the 12-month follow-up, information was collected over the phone by trained nurses via a standardized questionnaire. To offer a comprehensive overview of mental health difficulties among COVID-19 survivors, in line with investigations carried at the 12-month follow-up, information was collected with reference to common psychiatric disorders (i.e., depression, anxiety, and insomnia), cognitive impairment (i.e., a lack of concentration and focus), and somatic distress (i.e., fatigue) [8].

### 2.3. Statistical Analyses

Data analysis was performed by STATA 18, reporting absolute values and percentages in addition to comparing categorical variables across the time points (COVID-19 onset, 12- and 24-month follow-ups) via Cochran’s Q test for three time points or the McNemar test for two time points. Finally, after dichotomizing COVID-19 survivors based on whether they presented with post-COVID-19 neuropsychiatric symptoms, multivariable logistic regressions explored potential predictors of suffering from such symptoms based on statistical significance in univariable analysis, estimating the odds ratios (OR; 95% confidence interval, CI).

## 3. Results

### 3.1. Main Characteristics of the Whole Sample

Baseline data have been previously reported [8]. Out of 1.067 COVID-19 survivors initially screened, 230 patients completed the 24-month follow-up assessment. Most patients were middle-aged (41–60 years, 43%), native Italian (94.9%), females (53.5%), and presenting with a medical comorbidity (53.5%; Table 1).

### 3.2. Acute COVID-19 Presentation

Information on study sample characteristics in terms of acute COVID-19 presentation has been reported before [8]. Most COVID-19 patients had a symptomatic disease (92.6%) of mild severity (67.7%), with one in four patients presenting with moderate to critically severe COVID-19 (24.9%). Almost one-third of the sample required admission to hospital (28.7%), with a relatively low proportion of patients needing intensive care unit (ICU) support (5.2%). COVID-19 patients had a median in-hospital stay of 7 days (IQR 4–10).

### 3.3. Symptomatic Course over the 24-Month Follow-Up

One in three patients (36.1%) were still presenting with at least one symptom at the 24-month follow-up. Although a significant overall symptom reduction from the onset was observed (*p* < 0.001), symptom prevalence appeared to be substantially unchanged between the 12- and the 24-month follow-ups. Similar stable patterns were observed for most of the single symptomatic domains where, if a symptom reduction had been observed between the onset and the 12-month follow-up [3], no further reduction occurred over the subsequent 12 months. Additionally, in line with observations carried at the 12-month follow-up [3], neuropsychiatric symptoms were higher than at onset and were still the most frequently reported symptoms at the 24-month follow-up (symptoms of psychiatric disorders, 9.6%; a lack of concentration and focus, 25.2%; and fatigue, 14.4%). Additionally, a lack of concentration and focus was the only symptom that increased substantially even over the second year of observation, although just approaching significance (*p* = 0.071; Table 2).

### 3.4. Multivariable Logistic Models

Multivariable logistic models revealed that dyspnea at onset, but not care intensity, predicted symptoms of both psychiatric disorders (OR = 3.26, 95% CI = 1.22–8.70, and *p* = 0.019) and a lack of concentration and focus (OR = 3.17, 95% CI = 1.40–7.16, and *p* = 0.005) 24 months post-infection (Table 3 and 4). A significant association was also found for the number of comorbidities patients had at onset and the occurrence of a lack of concentration and focus at the 24-month follow-up (OR = 1.52, 95% CI = 1.12–2.08, and *p* = 0.008; Table 4). 

## 4. Discussion

COVID-19-induced neuropsychiatric symptoms may have plateaued 24 months post-infection, but they have not reduced. Rather, cognitive difficulties seem to have increased, with one out of four patients complaining about them at the 24-month follow-up. Such a pattern seems to be unique to neuropsychiatric symptoms, urging for studies specifically investigating predictors of post-COVID psychiatric syndrome to sustain the greatest possible recovery. Results indicate that patients with pre-existing vulnerabilities, as indicated by the number of medical comorbidities at COVID-19 onset, and those suffering from severe COVID-19, as suggested by the occurrence of dyspnea, possibly accounting for higher care intensity in the acute phase, are particularly susceptible to presenting with long-lasting neuropsychiatric symptoms.

The limitations of this study include the absence of a standardized approach to diagnose and treat post-COVID-19 neuropsychiatric symptoms, possibly jeopardizing comparability across studies. Additionally, as the study design does not allow disentangling the detrimental indirect effect of the pandemic, its contribution to the observed mental health aftereffects cannot be completely ruled out. Moreover, whether the detected effects are specific to COVID-19 or would have occurred among patients presenting with other infectious diseases remains to be tested. The strengths of the current study include the large sample, the longer follow-up when compared to most of the available evidence, and the extensive data gathering.

In conclusion, evidence from epidemiological and clinical studies provides converging and convincing proof that COVID-19 may result in neuropsychiatric sequalae. Consequently, the focus of research must move from investigating whether post-COVID neuropsychiatric syndrome exists to understanding the mechanisms involved and who is the most susceptible. In particular, future studies will have to focus on the inflammatory responses that COVID-19 may trigger in the brain, provoking damages, and subsequent symptom onset [9,10]. 

## Figures and Tables

**Table 1:** Baseline sociodemographic and clinical characteristics.

	*N* = 230
Gender, *n* (%) Female Male	123 (53.5) 107 (46.5)
Age Group, *n* (%) 18–40 41–60 >60	44 (19.1) 99 (43.0) 87 (37.8)
Ethnicity, *n*/*N* (%) Native Italian European	206/217 (94.9) 11/217 (5.1)
Comorbidities, Number, *n* (%) 0 1 2 3 ≥4	107 (46.5) 66 (28.7) 32 (13.9) 16 (7.0) 9 (3.9)
Comorbidities, *n*/*N* (%) Hypertension Obesity Diabetes Chronic respiratory disease ^ Cardiovascular disease * Liver disease Psychiatric disorders ° Renal impairment	47/226 (20.8) 29 (12.6) 15/229 (6.6) 8/229 (3.5) 4/229 (1.8) 7/229 (3.1) 3 (1.3) 0 (0)
Under Chronic Medication, *n*/*N* (%) Yes No	105/227 (46.3) 122/227 (53.7)

*n*, number; *N*, number as a denominator; ^, pulmonary disease: asthma, chronic obstructive pulmonary disease; *, cardiovascular disease: heart failure, ischemic heart disease, tachyarrhythmias, valvular heart disease, and venous thromboembolism; and °, depression, anxiety.

**Table 2:** Clinical trajectory over the 24-month follow-up period.

	Onset *n (%)*	12 Months *n (%)*	24 Months *n (%)*	*p*-Value	*p*-Value *
Overall Symptoms, Number 0 1 2 3 4 ≥5	27 (11.7) 35 (15.2) 49 (21.3) 31 (13.5) 43 (18.7) 45 (19.6)	116 (50.4) 48 (20.9) 33 (14.3) 16 (7.0) 4 (1.7) 13 (5.6)	147 (63.9) 20 (8.7) 17 (7.4) 15 (6.5) 14 (6.1) 17 (7.4)	<0.001	0.431
Mental-Health-Domain-Related Symptoms Symptoms of psychiatric disorders (depression, anxiety, insomnia) Lack of concentration and focus Fatigue	4 (1.7) 7 (3.0) 107 (46.5)	24 (10.4) 44 (19.1) 25 (10.9)	22 (9.6) 58 (25.2) 33 (14.4)	<0.001 <0.001 <0.001	0.860 0.071 0.217
Neurological-Domain-Related Symptoms Headache Neurological symptoms	75 (32.6) 16 (7.0)	14 (6.1) 5 (2.2)	10 (4.4) 4 (1.7)	<0.001 0.004	0.394 0.739
Anosmia/dysgeusia	135 (58.7)	24 (10.4)	18 (7.8)	<0.001	0.180
Rheumatological symptoms	30 (13.0)	35 (15.2)	33 (14.4)	0.756	0.768
Respiratory-Domain-Related Symptoms Dyspnoea URTI symptoms Cough Chest pain	74 (32.2) 28 (12.2) 96 (41.7) 7 (3.0)	20 (8.7) 3 (1.3) 7 (3.0) 7 (3.0)	16 (7.0) 6 (2.6) 10 (4.4) 5 (2.2)	<0.001 <0.001 <0.001 0.801	0.394 0.317 0.439 0.527
Cutaneous symptoms	6 (2.6)	3 (1.3)	6 (2.6)	0.500	0.180
Gastrointestinal symptoms	84 (36.5)	5 (2.2)	7 (3.0)	<0.001	0.527

* Comparison between 12 and 24 months; URTI, upper respiratory tract infection.

**Table 3:** Symptoms of psychiatric disorders (depression, anxiety, and insomnia) at the 24-month follow-up.

Risk Factors at Onset	Univariable Analysis		
	OR	95% CI	*p*-Value
Female gender	1.29	0.53, 3.14	0.580
Age group 18–40 41–60 >60	1 0.88 1.30	0.25, 3.09 0.38, 4.40	0.841 0.675
Ethnicity Native Italian European	1 0.88	0.11, 7.23	0.906
Co-morbidities, number	1.31	0.94, 1.83	0.114
Chronic medication	2.19	0.88, 5.45	0.091
Overall symptoms, number	1.15	0.93, 1.41	0.188
Symptoms of psychiatric disorders (depression, anxiety, and insomnia)	1.01	0.05, 19.37	0.995
Lack of concentration and focus	1.60	0.18, 13.96	0.669
Fatigue	1.43	0.59, 3.45	0.429
Headache	0.58	0.21, 1.64	0.303
Neurological symptoms	1.39	0.29, 6.54	0.680
Anosmia/dysgeusia	1.26	0.51, 3.13	0.621
Rheumatological symptoms	0.64	0.14, 2.90	0.566
Dyspnoea	4.32	1.72, 10.82	0.002
URTI symptoms	1.70	0.53, 5.46	0.370
Cough	1.77	0.73, 4.29	0.205
Chest pain	0.60	0.03, 10.80	0.727
Cutaneous symptoms	0.69	0.04, 12.70	0.804
Gastrointestinal symptoms	1.85	0.76, 4.47	0.172
Management Outpatient Ward Intensive care unit	1 3.08 5.13	1.18, 8.04 1.20, 22.0	0.022 0.028
Viral shedding	1.03	0.99, 1.07	0.204
**Risk Factors at Onset**	**Multivariable Analysis**		
	**OR**	**95% CI**	***p*-Value**
Dyspnoea	3.26	1.22, 8.70	0.019
Management Outpatient Ward Intensive care unit	1 2.24 2.70	0.82, 6.14 0.58, 12.60	0.116 0.208

URTI, upper respiratory tract infection.

**Table 4:** Lack of concentration and focus at the 24-month follow-up.

Risk Factors at Onset	Univariable Analysis		
	OR	95% CI	*p*-Value
Female gender	1.45	0.79, 2.66	0.227
Age group 18–40 41–60 >60	1 1.10 3.39	0.42, 2.87 1.35, 8.47	0.852 0.009
Ethnicity Native Italian European	1 2.53	0.74, 8.65	0.138
Co-morbidities, number	1.72	1.33, 2.22	<0.001
Chronic medication	3.41	1.80, 6.46	<0.001
Overall symptoms, number	1.23	1.06, 1.43	0.005
Symptoms of psychiatric disorders (depression, anxiety, and insomnia)	3.04	0.42, 22.05	0.272
Lack of concentration and focus	4.17	0.91, 19.23	0.067
Fatigue	2.11	1.15, 3.88	0.016
Headache	0.81	0.43, 1.56	0.536
Neurological symptoms	2.49	0.88, 7.01	0.085
Anosmia/dysgeusia	1.10	0.60, 2.01	0.768
Rheumatological symptoms	1.32	0.57, 3.07	0.519
Dyspnoea	4.22	2.25, 7.89	<0.001
URTI symptoms	1.78	0.77, 4.12	0.177
Cough	1.30	0.71, 2.37	0.391
Chest pain	1.19	0.23, 6.32	0.836
Cutaneous symptoms	0.22	0.01, 3.95	0.303
Gastrointestinal symptoms	1.76	0.96, 3.22	0.068
Management Outpatient Ward Intensive care unit	1 1.69 3.69	0.85, 3.36 1.12, 12.14	0.132 0.032
Viral shedding	1.03	0.99, 1.06	0.059
**Risk Factors at Onset**	**Multivariable Analysis**		
	**OR**	**95% CI**	***p*-Value**
Age group 18–40 41–60 >60	1 0.81 1.96	0.29, 2.31 0.67, 5.78	0.669 0.221
Co-morbidities, number	1.52	1.12, 2.08	0.008
Overall symptoms, number	1.09	0.87, 1.38	0.456
Fatigue	1.43	0.62, 3.27	0.398
Dyspnoea	3.17	1.40, 7.16	0.005
Management Outpatient Ward Intensive care unit	1 0.74 1.21	0.33, 1.68 0.31, 4.77	0.469 0.783

URTI, upper respiratory tract infection.

## Data Availability

Data available on request due to restrictions, e.g., privacy or ethical.
